# A Case of Natural Labor in a Negro-girl, Thirteen Years of Age

**Published:** 1855-04

**Authors:** W. S. Stoakley

**Affiliations:** Northampton County, Va.


*A case of Natural Labor in a Negro-girl, thirteen years of age.* By W. S. Stoakley, M. D., of Northampton County, Va.

I was called August, 1854, to see a negro girl, aged thirteen, suffering from colic. Upon examination, I was surprised to find in bed with her a child just two weeks old, which she claimed as hers.

The child was of the ordinary size, (larger, if anything, than the generality of children of that age,) hearty and hale. The mother was at the time troubled with a colic from eating watermelon, previous to which she had been perfectly well, not having had the slightest ailment of any kind since her confinement. On inquiry, I was told that the labor was a natural and easy one, and of the common duration. The pains were strong and effective. The placenta came away in fifteen minutes, and the womb returned as near as possible to its non-gravid condition, closing up with powerful contractions the bleeding mouths of the "rete-vasculosum," and instead of the torrents of blood, common on such occasions, gushing from the enlarged and anastomasing bloodvessels of the womb, nothing was seen but the normal quantity; the bounds of health were not overstepped. *If* she had been "under the shade which beechen boughs diffuse," nature would have "done her part," and the hand of art would have been an intruder. I placed my hand on her hypogastrium fourteen days after delivery, and found the uterus had returned to its unimpregnated state. I observed no flabbiness about the abdominal muscles. To all appearances she had regained her natural shape and symmetry.

